# Association between thyroid function and metabolic associated fatty liver disease: a systemic review and meta-analysis

**DOI:** 10.3389/fendo.2025.1660437

**Published:** 2025-10-29

**Authors:** Wei Xiong, Hua Fan, Jiake Tang, Qingwen Yu, Ziyi Xin, Ting Tang, Xiyun Rao, Lanlan Feng, Yongmin Shi, Xuhan Tong, Xinyan Fu, Xingwei Zhang, Mingwei Wang, Wentao Gan

**Affiliations:** ^1^ The First People’s Hospital of Jiande, Hangzhou, China; ^2^ Office of Research and Innovation, The First Affiliated Hospital of Henan University of Science and Technology, Luoyang, China; ^3^ Zhejiang Key Laboratory of Medical Epigenetics, Department of Cardiology, Affiliated Hospital of Hangzhou Normal University, Hangzhou Institute of Cardiovascular Diseases, Engineering Research Center of Mobile Health Management System & Ministry of Education, Hangzhou Normal University, Hangzhou, China; ^4^ Department of Cardiology, Hangzhou Lin’an Fourth People’s Hospital, Hangzhou, Zhejiang, China

**Keywords:** metabolic associated fatty liver disease, thyroid function, meta-analysis, systemic review, MAFLD

## Abstract

**Background:**

Metabolic dysfunction–associated fatty liver disease (MAFLD) is one of the most common chronic liver diseases. The relationship between MAFLD and thyroid function parameters remains controversial.

**Aim:**

This study aimed to explore the influence of metabolic parameters and thyroid dysfunction on the development of MAFLD and examine the relationship between them in different age groups.

**Methods:**

The PubMed, Embase, Web of Science, China National Knowledge Infrastructure(CNKI), and Cochrane Library databases were searched. Standardized mean difference (SMD) and odds ratio with a 95% confidence interval (CI) were calculated.

**Results:**

A total of 36 studies involving 198,254 participants were eligible. Compared with controls, the patients with MAFLD had significantly higher thyroid-stimulating hormone (TSH) levels (MAFLD vs controls: SMD = 0.02, 95% CI = 0.01–0.03); significantly higher free triiodothyronine levels (MAFLD vs controls: SMD = 0.19, 95% CI = 0.18–0.20); significantly lower free thyroxine levels (MAFLD vs controls: SMD = −0.41, 95% CI = −0.42 to −0.40); significantly higher total triiodothyronine levels (MAFLD vs controls: SMD = 0.26, 95% CI = 0.23–0.29); and significantly lower total thyroxine levels (MAFLD vs controls: SMD = −0.10, 95% CI = −0.13 to −0.07).

**Conclusions:**

The TSH level may be an important risk factor for the occurrence and development of MAFLD. The relationship between them is influenced by age.

## 1 Introduction

Metabolic dysfunction–associated fatty liver disease (MAFLD) encompasses a range of liver diseases closely related to abnormal metabolic disorders. It is considered a hepatic manifestation of the metabolic syndrome (1). MAFLD is characterized by hepatic steatosis (detected by imaging techniques, blood biomarkers/scores, or liver histology) associated with type 2 diabetes mellitus (T2DM) and overweight/obesity, regardless of alcohol intake or excluding other causes of chronic liver disease (2). The introduction of the new concept of MAFLD aims to include metabolic dysfunction as a diagnostic criterion and covers a larger and more diverse population than non-alcoholic fatty liver disease (NAFLD) (3). A meta-analysis involving 3.3 million people revealed that the global prevalence of MAFLD was 38.77% (4). Compared with the general population, patients with MAFLD experience higher liver-related morbidity or mortality and are strongly associated with extrahepatic diseases such as cancer, cardiovascular events, stroke, chronic kidney disease, and obstructive sleep apnea (5–8).

The rationale for MAFLD is to include metabolic dysfunction as a diagnostic criterion, including abdominal obesity and T2DM. The thyroid gland regulates body weight, lipid-like substance metabolism, and insulin resistance. Therefore, thyroid hormone may be closely related to the occurrence and development of MAFLD (9). However, the relationship between MAFLD and thyroid function still remains controversial. Three published meta-analyses have explored the relationship between NAFLD and thyroid function, but their findings were inconsistent (10–12). The patients with NAFLD/NASH had significantly higher thyroid-stimulating hormone (TSH) levels than controls in adults. Previous studies found no significant difference in thyroid hormone levels between patients with NAFLD and non-NAFLD. This might be mainly attributed to the heterogeneity of sample sizes and patient characteristics. MAFLD covers a much broader spectrum, and no studies demonstrating an association between MAFLD and thyroid function parameters have been reported. Therefore, this study conducted a new systematic review and meta-analysis, including a lot of research and exploration of several thyroid function parameters [free triiodothyronine (FT3), free thyroxine (FT4), total triiodothyronine (TT3), total thyroxine (TT4), and TSH levels].

## 2 Methods

### 2.1 Protocol

This systematic review and meta-analysis was conducted based on the meta-analysis of observational studies following epidemiology guidelines.

### 2.2 Eligibility criteria

The inclusion criteria were as follows: (1) original studies that explored the association between MAFLD, NAFLD, and NASH with thyroid function; (2) studies independent of sex and age; and (3)studies in which MAFLD was diagnosed by histology (liver biopsy), imaging modalities (such as ultrasonography, CT, or MRI), or validated biochemical indices. The exclusion criteria were as follows: (1) reviews and meta-analyses, (2) irrelevant pieces of literature, (3) duplicate studies, (4) experimental studies, (5) editorials/comments and case reports, and (6) original studies including patients with MAFLD/NAFLD/NASH but with competing etiologies for steatosis and coexisting causes for other chronic liver diseases, such as significant alcohol consumption, hepatitis C, and medications.

### 2.3 Search strategy

We searched the PubMed, Embase, Web of Science, CNKI, and Cochrane Library databases from the inception dates to March 1, 2025, with no restrictions on language. Conference abstracts were hand searched to identify potentially eligible studies. The search strategy was as follows: (“MAFLD” [All Fields] OR “Metabolic associated fatty liver disease” [All Fields]) OR (“NASH” [All Fields] OR “Nonalcoholic Steatohepatitis” [All Fields]) OR (“NAFLD” [All Fields] OR “Non-alcoholic Fatty Liver Disease” [All Fields]) AND (“thyroid” [All Fields] OR “thyroid-stimulating hormone” [All Fields] OR “free thyroxine” [All Fields] OR “free triiodothyronine” [All Fields] OR “total triiodothyronine” [All Fields] OR “total thyroxine” [All Fields]).

### 2.4 Study selection

Two reviewers (J.T. and C.C.) independently screened the studies based on the titles and abstracts. If the studies were potentially fit, full texts were further retrieved and screened. Disagreements were resolved by consensus.

### 2.5 Data extraction

Two reviewers (J.T. and C.C.) extracted all the data independently. We extracted the following data from each study: study characteristics, first author, year of publication, country, study design, study population, number of participants, age, MAFLD diagnosis, and thyroid function parameters (TSH, FT3, FT4, TT3, and TT4). If necessary, the reviewers contacted for relevant data.

If some data were expressed as mean with a 95% confidence interval (CI) alone, the methods provided by the Cochrane Handbook were used to calculate the standard deviation (SD). The formula used is as follows:


$$\text{SD}=\sqrt{N}\times \left(\text{upper }\mathrm{limit}-\text{lower limit}\right)/3.92$$

If some data were expressed as median with range or interquartile range alone, the methods provided by Wan et al. (13) and Luo et al. (14) were used to estimate the mean and SD. All formulas are available on the website http://www.comp.hkbu.edu.hk/∼xwan/MedianRange.html.

### 2.6 Statistical analysis

The association of MAFLD/NASH/NAFLD with thyroid function parameters (including FT3, FT4, TT3, TT4, and TSH) was assessed. For continuous data, standardized mean difference (SMD) with 95% CI was calculated using the inverse variance statistical method. For dichotomous variable data, the odds ratio (OR) with 95% CI was calculated using the Mantel–Haenszel statistical method. The data were pooled using a random-effects model to obtain a more conservative effect estimate. The *I* ^2^ statistic and *Q* test were used to measure the heterogeneity across the included studies, where *I* ^2^ >50% and *P* value <0.1 were considered to be of significant heterogeneity. If more than five eligible studies were included in a meta-analysis, the publication bias was assessed using funnel plots. All statistical analyses were performed using Review Manager version 5.2 software.

## 3 Results

### 3.1 Study selection

**Figure 1:**
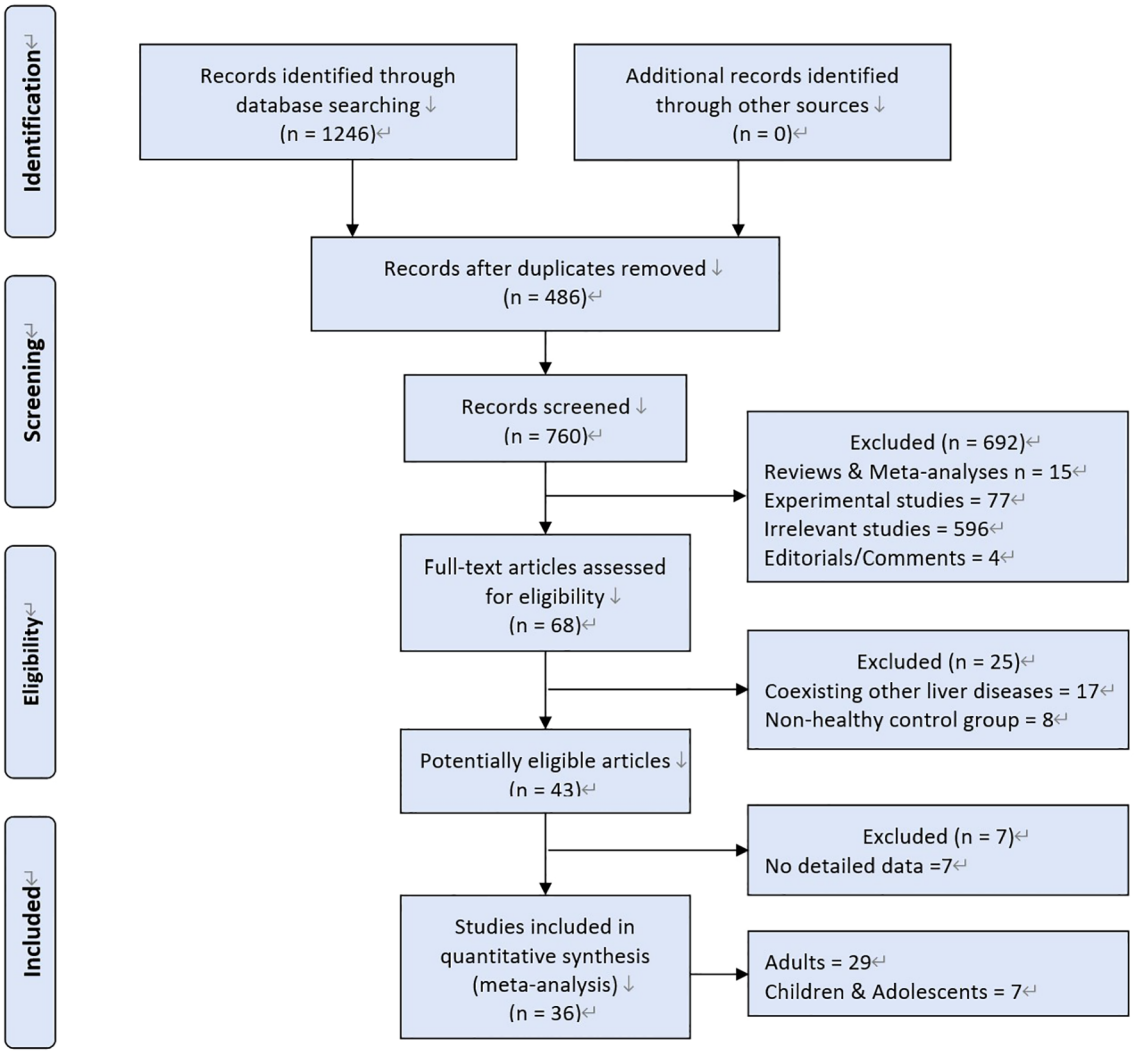
Study screening flow chart.

A total of 1246 studies were retrieved, 36 studies were eligible (**Figure 1**). MAFLD was diagnosed by hepatic steatosis (including imaging techniques, blood biomarkers/scores, or liver histological tests). Patients without MAFLD rather than healthy individuals were randomly selected as controls. Ultimately, 198,254 participants were included in the quantitative analysis.

### 3.2 Study characteristics

The characteristics of the 36 included studies are depicted(**Table 1**). Among these, 7 were cohort studies (15–21), 25 were cross-sectional studies (22–46), and 4 were case–control studies (47–50). These clinical trials were conducted globally: 6 in Europe [Germany (23, 26), Italy (18, 47), Netherlands (30), and Spain (39)], 28 in Asia (5 in Turkey (15–17, 27, 29), 2 in South Korea (24, 36), 1 in India (20), 1 in Iran (22), 19 in China [19, 21, 25, 28, 31–35, 37, 38, 40–46)], 2 in Africa [Egypt (48, 50)], and 1 in the United States (49). MAFLD was diagnosed by different methods: through liver biopsies in 5 studies (18, 37, 47, 49, 50), through computed tomography (CT) in 1 study (41), and through ultrasound technology in 31 studies. All the 36 included studies reported detailed data regarding thyroid function parameters (**Table 2**). All studies reported detailed data on the TSH parameter, 22 studies reported data on the FT3 parameter, 28 on the FT4 parameter, and 6 on the TT3 and TT4 parameters.

**Table 1:** Characteristics of included studies.

First author(Year)	Country	Study design	No. participants	Enrollment period	Diagnosis of MALFD	Thyroid function parameters
Ambrosio 2021	Italy	Cross-sectional study	53	2014−2016	liver biopsy	TSH, FT3, FT4
Basarir 2021	Turkey	Cross-sectional study	208	2015.02−2017.02	ultrasonography	TSH, FT4
Berg 2017	Netherlands	Cross-sectional study	20289	NA	ultrasonography	TSH, FT3, FT4
Bilgin 2014	Turkey	Cross-sectional study	120	2010.04−2011.06	ultrasonography	TSH, FT3, FT4
Carulli 2013	Italy	Case-control study	69	2001−2008	liver biopsy	TSH, FT3, FT4
Chao 2021	China	Cross-sectional survey	55448	2017.01−2019.02	ultrasonography	TSH, FT3, FT4
Chen 2019	China	Cross-sectional study	1838	2013.04−2016.09	ultrasonography	TSH, FT3, FT4
Du 2021	China	Cross-sectional study	1422	2014.12−2019.10	ultrasonography	TSH, FT3, FT4
Escudé 2020	Spain	Cross-sectional study	10116	2009−2013	ultrasonography	TSH
Eshraghian 2013	Iran	Cross-sectional study	832	2011.09−2012.09	ultrasonography	TSH, FT3, FT4
Fan 2022	China	Cross-sectional study	18427	2015.01−2021.12	ultrasonography	TSH, FT3, FT4, TT3, TT4
Fatma 2016	Turkey	Cross-sectional study	61	2014.7−2015.01	ultrasonography	TSH, FT3, FT4
Feng 2019	China	Cross-sectional study	589	2015.01−2017.12	ultrasonography	TSH, FT3, FT4
Gu 2021	China	Cross-sectional study	6462	2013−2019.	ultrasonography	TSH, FT3, FT4
Guo 2021	China	Cross-sectional study	3497	2017.11−2019.12	ultrasonography	TSH, FT3, FT4
Gupta 2020	India	Cross-sectional study	100	2018.09−2019.10	ultrasonography	TSH, TT3, TT4
Kaltenbach 2017	Germany	Cross-sectional study	332	2000.02−2001.05	ultrasonography	TSH, TT3, TT4
Kim 2022	Korea	Cross-sectional study	1589	2013.01−2015.12	ultrasonography	TSH, FT4
Lee 2015	Korea	Cohort study	18544	2008.01−2012.12	ultrasonography	TSH, FT4
Li 2022	China	Cross-sectional study	129	2017.01−2021.01	liver biopsy	TSH, FT3, FT4
Liang 2018	China	Cross-sectional study	32573	2016.01−2016.12	ultrasonography	TSH, FT4
Liu 2014	China	Cross-sectional study	2576	2013.01−2013.11	ultrasonography	TSH, FT3, FT4
Liu 2018	China	Cross-sectional study	1773	2015.01−2015.12	ultrasonography	TSH, FT3, FT4
LIU 2019	China	Cross-sectional study	15660	2013.01−2018.10	ultrasonography	TSH, FT3, FT4
Ludwig 2015	Germany	Cross-sectional study	1276	NA	ultrasonography	TSH, TT3, TT4
Moustafa 2009	Egypt	Case-control study	33	NA	ultrasonography	TSH, TT3, TT4
Naguib 2021	Egypt	Case-control study	100	NA	liver biopsy	TSH, FT4
Pagadala 2012	USA	Case-control study	663	2006.10−2009.06	Liver biopsy	TSH
Pan 2019	China	Cross-sectional study	129	2013−2014	ultrasonography	TSH
Sert 2013	Turkey	Cross-sectional study	100	2011.04−2012.04	ultrasonography	TSH, FT4
Shao 2022	China	cross-sectional study	115	2019.01−2021.10	computed tomography (CT)	TSH, FT3, FT4, TT3, TT4
Tao 2014	China	cross-sectional study	739	2012.09−2013.02	ultrasonography	TSH, FT3, FT4
Torun 2014	Turkey	Cross-sectional study	72	2011.01−2013.02	ultrasonography	TSH, FT3, FT4
Xu 2011	China	Cross-sectional study	878	2008.01−2008.12	ultrasonography	TSH, FT3, FT4
ZHANG 2012	China	Cross-sectional study(female)	777	2010.01 to 2010.04	ultrasonography	TSH
		Cross-sectional study (male)	545	2010.01 to 2010.04	ultrasonography	TSH
Zhang 2022	China	Cross-sectional study	120	2016.01 to 2022.09	ultrasonography	TSH, FT3, FT4

**Table 2:** Table of thyroid function indicators.

First author(Year)	Groups	Age	No. Pts	TSH (mIU/l)	FT3 (pmol/l)	FT4 (pmol/l)	TT3 (nmol/l)	TT4 (nmol/l)
Ambrosio 2021	NAFLD	58±28.9	28	2.38±26.89	5.2±2.07	16.3±11.93	NA	NA
	NO-NAFLD	52±32.59	25	2.17±7.82	5.6±2.29	16.2±4.59	NA	NA
Basarir 2021	NAFLD	12.6±2.6	94	2.5±1.3	NA	13±3.9	NA	NA
	NO-NAFLD	10.8±2.7	114	2.3±1.2	NA	13±3.9	NA	NA
Berg 2017	NAFLD	46±8.89	4274	2.04±0.84	5.3±0.52	15.4±1.85	NA	NA
	NO-NAFLD	42±11.85	16015	2.01±0.86	5.2±0.52	15.7±1.78	NA	NA
Bilgin 2014	NAFLD	12.68±2.13	80	3.04±1.43	6.67±1.05	16.34±2.45	NA	NA
	NO-NAFLD	11.90±2.52	40	2.75±1.25	5.71±0.79	17.63±2.06	NA	NA
Carulli 2013	NAFLD	44.41 ±11.43	44	2.21±0.95	5.21±0.69	13.44±2.95	NA	NA
	NO-NAFLD	44.40 ±11.38	25	1.67±0.74	5.1±0.51	12.83±2.42	NA	NA
Chao 2021	NAFLD	48.8 ±9.79	19003	1.85±1.55	4.13±1.29	26.13±1.92	NA	NA
	NO-NAFLD	46.0 ±10.7	36445	1.88±1.67	3.99±1.39	28.34±2	NA	NA
Chen 2019	NAFLD	57.3±6.4	914	2.64±0.83	4.96±0.51	15.54±1.72	NA	NA
	NO-NAFLD	56.8±6.6	924	2.61±0.81	4.85±0.47	15.56±1.68	NA	NA
Du 2021	MAFLD	53.07±13.32	836	2.05±1.17	4.21±1.5	16.42±7.46	NA	NA
	NO-MAFLD	57.28±12.13	586	2.01±1.21	4.5±1.01	16.69±10.95	NA	NA
Escudé 2020	NAFLD	60±10	6790	2.3±1.9	NA	NA	NA	NA
	NO-NAFLD	61±10	3326	2.2±1.9	NA	NA	NA	NA
Eshraghian 2013	NAFLD	48.20±12.82	127	2.02±1.35	3.8±0.73	15.84±2.76	NA	NA
	NO-NAFLD	36.97 ±18.76	705	2.29±1.47	4.09±1.64	16.51±5.63	NA	NA
Fan 2022	MAFLD	51.09±15.78	4952	1.98±0.9	4.66±0.5	13.98±1.87	1.63±0.24	94.39±15.91
	NO-MAFLD	45.59±16.13	13475	1.95±0.9	4.5±0.51	14.11±1.87	1.56±0.24	95.53±15.84
Fatma 2016	NAFLD	49.78 ±12.77	36	1.71±0.96	4.39±0.8	9.27±2.57	NA	NA
	NO-NAFLD	46.52 ±13.80	25	1.5±0.83	4.48±0.52	10.3±2.06	NA	NA
Feng 2019	NAFLD	64.42±13.68	217	2.46±0.99	4.76±0.56	16.64±1.94	NA	NA
	NO-NAFLD	72.60±14.56	372	1.76±0.75	4.25±0.64	16.86±1.99	NA	NA
Gu 2021	MAFLD	51.50±7.90	1675	1.93±0.87	5.05±0.62	15.73±2.21	NA	NA
	NO-MAFLD	51.0±8.48	4787	2.05±0.9	4.9±0.62	15.47±2.21	NA	NA
Guo 2021	NAFLD	49.49 ± 9.86	2173	2.2±0.84	5.08±0.59	17.06±1.95	NA	NA
	NO-NAFLD	49.28 ±10.46	1324	2.25±0.83	4.77±0.48	16.96±1.98	NA	NA
Gupta 2020	NAFLD	10.52±2.2	62	3.93±1.4	NA	NA	2.22±0.38	82.27±31.18
	NO-NAFLD	9.32±2.4	38	4.47±1.8	NA	NA	8.49±2.6	82.5±25.74
Kaltenbach2017	NAFLD	14.1±1.9	99	2.8±1.1	NA	NA	1.7±0.4	103±18.0
	NO-NAFLD	13.9±1.8	233	2.5±1.4	NA	NA	1.6±0.3	103±18
Kim 2022	NAFLD	55±7.41	378	2.3±1.78	NA	15.47±1.95	NA	NA
	NO-NAFLD	56 ±7.41	1211	2.21±1.13	NA	15.6±1.95	NA	
Lee 2015	NAFLD	39.2±5.9	2348	2.3±4.3	NA	16.47±2.06	NA	NA
	NO-NAFLD	37.8±5.7	16196	2.3±2.6	NA	16.21±1.93	NA	NA
Li 2022	NAFLD	51.5±14.07	40	2.87±1.27	5.11±0.49	15.67±2.18	NA	NA
	NO-NAFLD	43.0 ±20.74	89	2.11±1.16	4.74±0.78	15.75±2.23	NA	NA
Liang 2018	NAFLD	48.8±13.4	5183	2.95±1.49	NA	16.32±2.01	NA	NA
	NO-NAFLD	43.23±13.7	27390	2.86±1.48	NA	16.47±1.93	NA	NA
Liu 2014	NAFLD	46.90± 9.41	988	2.13±0.9	5.12±0.58	16.41±2.04	NA	NA
	NO-NAFLD	44.42±10.74	1588	2.2±0.93	4.84±0.58	16.18±2.06	NA	NA
Liu 2018	NAFLD	50.42±8.90	638	1.67±0.88	4.63±0.55	12.79±1.33	NA	NA
	NO-NAFLD	49.30±11.36	1135	1.58±0.93	4.43±0.53	12.71±1.48	NA	NA
LIU 2019	NAFLD	49.3±10.2	7095	1.65±1.21	4.51±0.7	13.07±1.98	NA	NA
	NO-NAFLD	47.3±11.9	8565	1.6±1.1	4.35±0.68	13.27±2	NA	NA
Ludwig 2015	NAFLD	47.7 ± 11.5	349	1.8±1.4	NA	NA	1.6±0.3	83.2±15.6
	NO-NAFLD	38.0 ± 12.1	927	1.8±3.5	NA	NA	1.6±0.3	92±17.4
Moustafa 2009	NAFLD	55±4.6	13	2.1±0.75	NA	NA	1.84±0.27	100.39±14.8
	NO-NAFLD	51±9.3	20	1.75±0.9	NA	NA	1.89±0.38	94.72±15.44
Naguib 2021	NAFLD	47.9 ±3	50	3.3±1.2	NA	13±3.9	NA	NA
	NO-NAFLD	45.7 ±4	50	2.2±0.8	NA	15.6±3.9	NA	NA
Pagadala 2012	NAFLD	50.4±11.1	233	2.27±1.33	NA	NA	NA	NA
	NO-NAFLD	51.0 ±14.1	430	1.7±0.67	NA	NA	NA	NA
Pan 2019	NAFLD	12.47 ± 3.24	17	6.17±9.29	NA	NA	NA	NA
	NO-NAFLD	11.97 ± 3.39	112	4.01±3.4	NA	NA	NA	NA
Sert 2013	NAFLD	13.13 ± 1.26	58	3.56±1.34	NA	15.44±1.93	NA	NA
	NO-NAFLD	13.65 ± 1.4	42	1.77±0.63	NA	16.09±1.93	NA	NA
Shao 2022	NAFLD	44±15.56	81	1.77±0.87	5.13±0.67	11.38±1.97	1.52±0.27	105.3±16.12
	NO-NAFLD	34±12.59	34	1.82±1.13	4.93±0.44	13.01±1.76	1.54±0.23	106.62±19.75
Tao 2014	NAFLD	48.9± 8.4	196	1.96±1.07	4.86±0.73	16.87±1.77	NA	NA
	NO-NAFLD	47.5± 8.1	543	1.81±0.92	4.88±0.57	17.5±1.87	NA	NA
Torun 2014	NAFLD	12.2 ±3.6	28	4.2±3.9	5.7±1.1	12.9±4.4	NA	NA
	NO-NAFLD	12.8 ±1.7	44	2.2±1.43	5.4±0.9	13.2±2.3	NA	NA
Xu 2011	NAFLD	71.2±3.8	227	2±0.93	4.6±0.44	11.12±1.43	NA	NA
	NO-NAFLD	71.9±4.2	651	1.82±0.88	4.58±0.49	11.58±1.47	NA	NA
ZHANG 2012(1)	NAFLD	NA	129	3.26±1.33	NA	NA	NA	NA
(female)	NO-NAFLD	NA	648	2.98±1.25	NA	NA	NA	NA
ZHANG 2012(2)	NAFLD	NA	137	2.56±1.16	NA	NA	NA	NA
(male)	NO-NAFLD	NA	408	2.34±1.08	NA	NA	NA	NA
Zhang 2022	MAFLD	57±8.89	47	1.78±1.07	5.04±0.58	17±2.59	NA	NA
	NO-MAFLD	55±12.59	73	1.96±1.33	4.68±0.59	16.9±2.89	NA	NA

NA, not applicable.

#### 3.2.1 Thyroid-stimulating hormone

**Figure 2:**
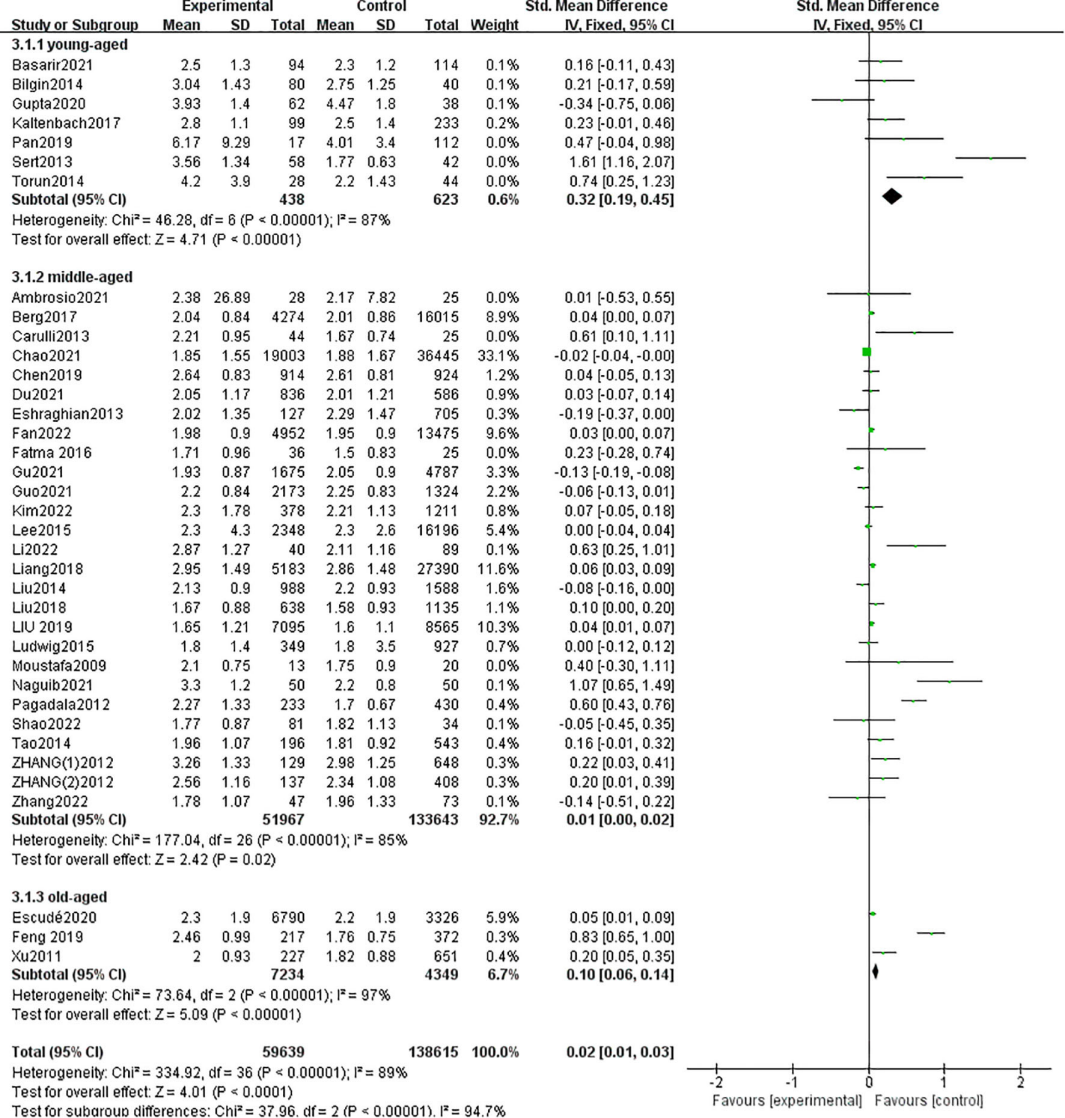
Forest plot comparing the TSH between MAFLD and no-MAFLD.

**Figure 3:**
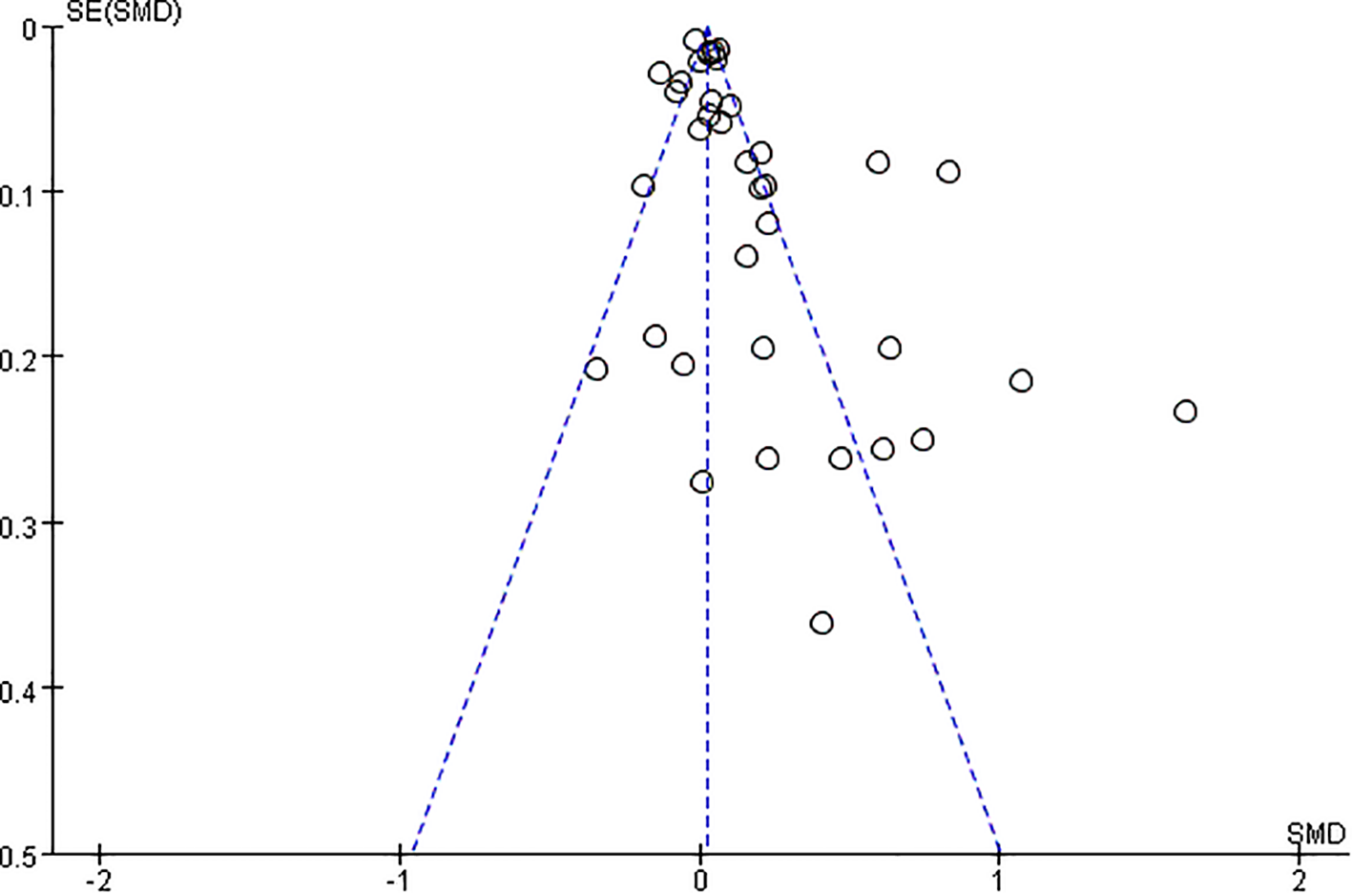
Funnel plot comparing the TSH between MAFLD and no-MAFLD.

A total of 36 studies with 198,254 patients were included in the meta-analysis. The patients with MAFLD had significantly higher levels of TSH than controls (SMD = 0.02; 95% CI = 0.01, 0.03; *P* < 0.001) (**Figure 2**). Heterogeneity was statistically significant (*I* ^2^ = 89%, *P* < 0.001). The publication bias was not statistically significant (**Figure 3**). Meanwhile, it was found that compared with the controls, the patients with MAFLD had significantly higher levels of TSH in the children/youth group according to the age subgroup analysis (SMD = 0.32, 95% CI = 0.19, 0.045, *P* < 0.001); no significant difference was observed in the level of TSH in the middle-aged group (SMD = 0.01, 95% CI = 0.00, 0.02, *P* = 0.02); and significantly higher levels of TSH was observed in the elderly group (SMD = 0.10, 95% CI = 0.06, 0.14, *P* < 0.001).

#### 3.2.2 Free triiodothyronine

**Figure 4:**
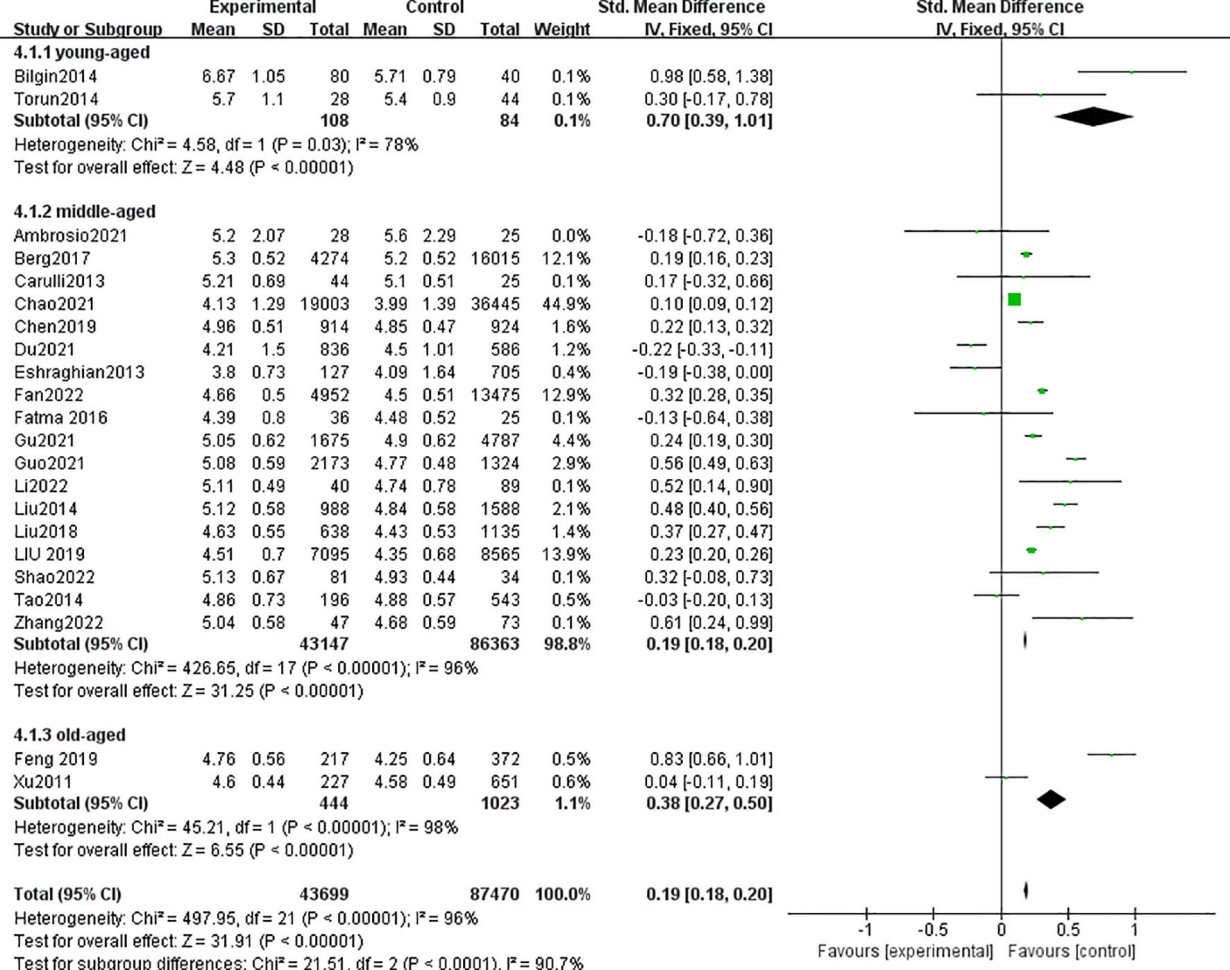
Forest plot comparing the FT3 between MAFLD and no-MAFLD.

A total of 22 studies with 121,169 patients were included in the meta-analysis. The patients with MAFLD had significantly higher levels of FT3 than controls (SMD = 0.19, 95% CI = 0.18, 0.20, *P* < 0.001) (**Figure 4**). Heterogeneity was statistically significant (*I* ^2^ = 89%, *P* < 0.001). Compared with the controls, the patients with MAFLD had significantly higher levels of FT3 in the children/youth group (SMD = 0.70, 95% CI = 0.39, 1.01, *P* < 0.001); the middle-aged group (SMD = 0.19, 95% CI = 0.18, 0.2, *P* < 0.001); and the elderly group (SMD = 0.38, 95% CI = 0.27, 0.50, *P* < 0.001).

#### 3.2.3 Free thyroxine

**Figure 5:**
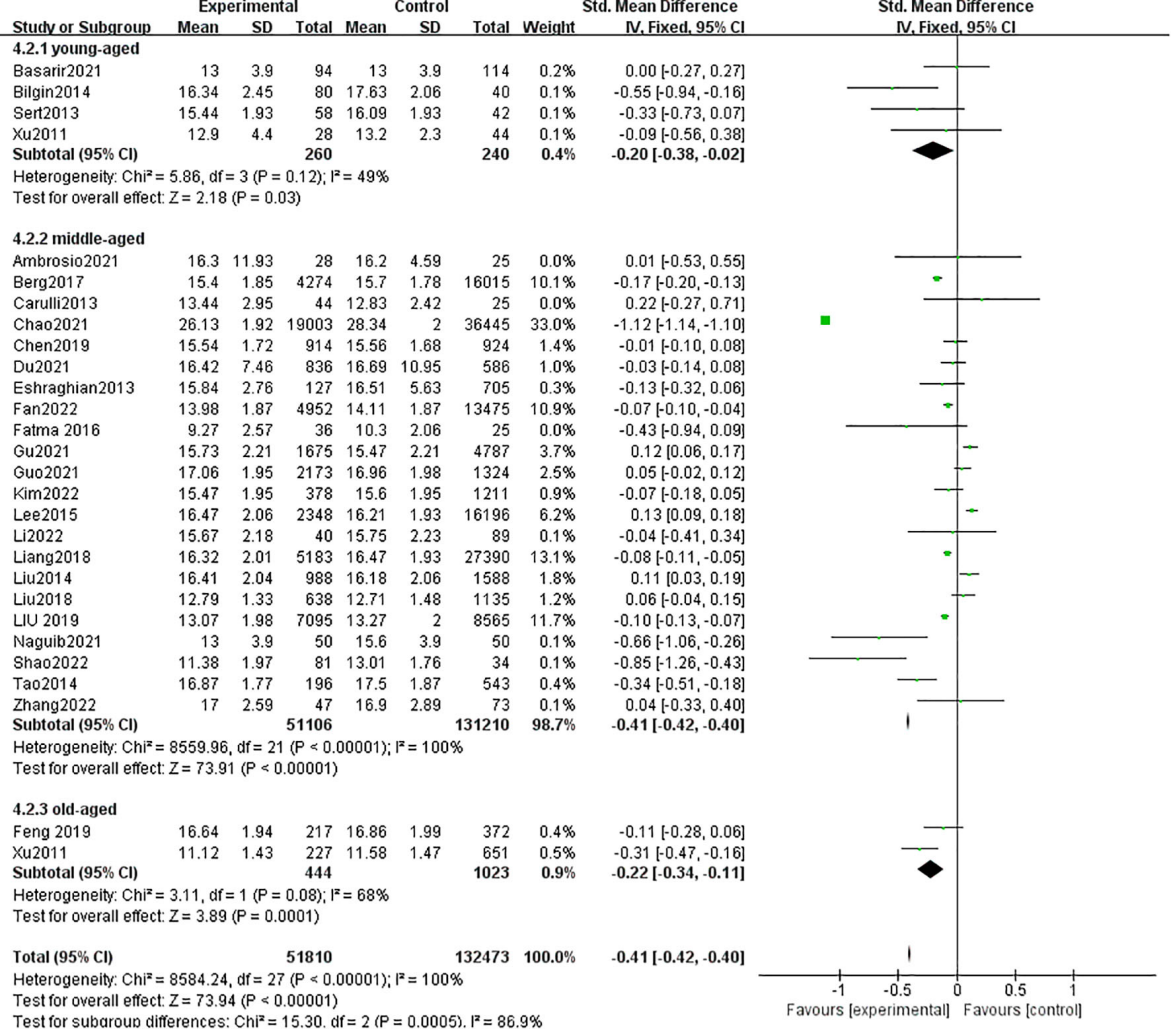
Forest plot comparing the FT4 between MAFLD and no-MAFLD.

A total of 28 studies with 184,283 patients were included in the meta-analysis. The patients with MAFLD had significantly lower levels of FT4 than controls (SMD = −0.41, 95% CI = −0.42, −0.40, *P* < 0.001) (**Figure 5**). Heterogeneity was statistically significant (*I* ^2^ = 100%, *P* < 0.001). Compared with controls, the patients with MAFLD had significantly lower levels of FT4 in the children/youth group (SMD = −0.20, 95% CI = −0.38, −0.02, *P* < 0.03); the middle-aged group (SMD = −0.41, 95% CI = −0.42, −0.40, *P* < 0.001); and the elderly group (SMD = −0.22, 95% CI = −0.34, −0.11, *P* < 0.001).

#### 3.2.4 Total triiodothyronine

**Figure 6:**
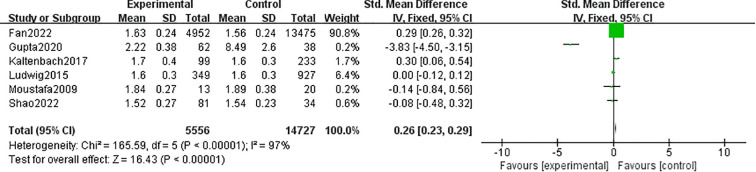
Forest plots comparing the TT3 between MAFLD and no-MAFLD.

A total of 6 studies with 20,283 patients were included in the meta-analysis. The patients with MAFLD had significantly higher levels of TT3 than controls (SMD = 0.26, 95% CI = 0.23, 0.29, *P* < 0.001) (**Figure 6**). Heterogeneity was statistically significant (*I* ^2^ = 97%, *P* < 0.001).

#### 3.2.5 Total thyroxine

**Figure 7:**
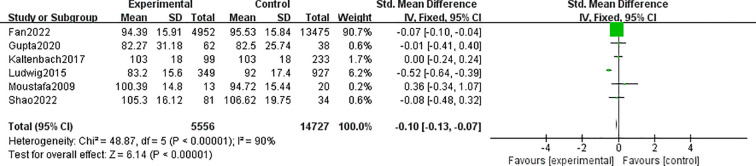
Forest plots comparing the TT4 between MAFLD and no-MAFLD.

A total of 6 studies involving 20,283 patients were included in the meta-analysis. The patients with MAFLD had significantly lower levels of TT3 than controls (SMD = −0.10; 95% CI = −0.13, −0.07; *P* < 0.001) (**Figure 7**). Heterogeneity was statistically significant (*I* ^2^ = 90%, *P* < 0.001).

## 4 Discussion

A total of 36 studies with 198,254 participants were included in the systematic review and meta-analysis. The findings revealed the following. (1) Increased TSH levels were significantly associated with a higher risk of MAFLD. However, no significant difference in the TSH level was found between the patients with MAFLD and the controls in the middle-aged population. In the adolescent and elderly population, the TSH level of patients with MAFLD significantly differed from that of the controls. (2) The increased levels of FT3 and TT3 were significantly associated with the risk of MAFLD in all populations. (3) The decreased levels of TT4 and FT4 were significantly associated with the risk of MAFLD in all populations.

The “second strike” hypothesis has been widely considered to be the classical pathogenesis of MAFLD. The progressive accumulation of lipids in the cytoplasm of hepatocytes triggers a cytotoxic event that triggers an inflammatory response. The liver, being the central organ regulating endocrine function and maintaining triglyceride metabolism, plays a crucial role. Abnormal triglyceride accumulation in hepatocytes forms fatty liver. Some studies have shown a positive correlation between TSH and triglyceride concentrations (51, 52), even within the normal reference range (53). TSH might play a key role in regulating triglycerides and cholesterol, thus promoting the development of MAFLD (54).

In terms of pathophysiology, the presence of thyroid hormones can contribute to the development of MAFLD through multiple pathways. Thyrotropin hormone regulates the thyrotropin receptor (TSHR) through highly specific interactions with thyroxine. TSHR is expressed primarily on the cell membrane of thyroid follicular cells and is also expressed to varying degrees in numerous extrathyroidal tissues and cells, such as liver, kidney, bone, adipose tissue, and fibroblasts (55, 56). The binding of TSH to TSHR on hepatocytes can increase triglyceride levels, leading to hepatic steatosis. Thus, the potential mechanism of TSH-induced hepatic triglyceride accumulation involves the binding of TSH to its receptor TSHR, which then triggers hepatic Sterol Regulatory Element Binding Protein-1c(SREBP-1c)activity via the cAMP/PKA/PPARa pathway, resulting in reduced AMPK activity and increased expression of genes associated with insulin resistance and adipogenesis (57). Meanwhile, another study showed that TSH could be involved in intrahepatic lipolysis by the activation of autophagy and β-oxidation of fatty acids (58). Thus, elevated TSH levels indicated hypothyroidism and reduced activity of hepatic lipase, leading to fat cell accumulation in hepatocytes (59). A study demonstrated that foreign bodies from TSH-stimulated hepatocytes increased and showed specific protein profile changes, many of which were involved in metabolism, signal transduction, apoptosis, and inflammation (60). TSH is also associated with the regulation of microrne (61). Adipokine levels were altered in patients with hypothyroidism (62). Recently, a high TSH level was significantly correlated with NASH in individuals with normal thyroid function and NAFLD confirmed by biopsy (63). In this study, TSH increased the risk of MAFLD significantly. However, the results of this study were controversial. The subgroup analysis in this study showed no significant association between TSH and MAFLD in the middle-aged population, but found an association between TSH and MAFLD in adolescents and older adults.

A feedback loop exists between TSH and thyroid gland in the body. An increase in the level of TSH usually suggests a low thyroid function (i.e., hypothyroidism) (12). Ferrandino et al. demonstrated the development of NAFLD in mice with mild hypothyroidism without the downregulation of hepatic TH signaling or reduced hepatic lipid utilization. This condition led to an increased shuttling of fatty acids (FAs) to the liver, where they were esterified and accumulated as triglycerides. On the contrary, mice with severe hypothyroid exhibited a downregulation of hepatic TH signaling and a severe inhibition of adipose tissue lipolysis, which reduced the delivery of FAs to the liver. The resulting lack of triglyceride esterification substrates protected mice with severely hypothyroid against NAFLD (64). We speculated that MAFLD was induced by insufficient release of TSH in adolescents and the elderly, when the thyroid was immature or in the process of degeneration, respectively. In adults, the pituitary-thyroid axis may be mature enough to maintain adequate TSH secretion, which helps regulate lipid metabolism in the liver.The association between TSH and MAFLD might be influenced by age. Selin et al. demonstrated that children with metabolic disease had lower levels of FT4 and higher levels of TSH than normal children (65). Gu et al. found that FT3 was a higher risk factor for NAFLD than those without NAFLD, while FT4 and TSH were not significantly correlated with NAFLD in middle-aged and elderly people (age >40) (46). This also explained why such a wide divergence was observed in the existing studies, with some suggesting no association and others suggesting an association. This might depend on the age distribution of the population included in previous studies and the differences in TSH concentrations.

The thyroid hormone receptor (TR) is of two types, TRα and TRβ, and it is the main receptor in the liver (66). Studies have shown that M1 macrophages polarizably induce secreted phosphoprotein 1 (SPP1) secretion and downregulate hepatocyte TRβ in a paracrine manner, exacerbating lipid deposition in the liver and compensating for increased serum TSH. Increased levels of TSH can lead to SPP1 secretion by M1 macrophages. The positive feedback interaction between the thyroid and liver may play a significant role in maintaining and amplifying the pathological process of MAFLD (67). Therefore, TRβ agonists have been investigated as potential therapies for serum dyslipidemia and MAFLD (68). Chaves et al. demonstrated that mutations in the THR-beta gene of TR can induce that signal transduction damage in the liver, leading to hepatic steatosis. This indicates the influence of thyroid hormones on lipid metabolism in the liver (69). Our findings supported the effect of thyroxine on MAFLD. This study found that elevated levels of FT3 and TT3 were significantly associated with the risk of MAFLD, and decreased levels of TT4 and FT4 were significantly associated with the risk of MAFLD. Further clinical studies are needed to investigate whether TSH affects FT3, FT4, TT3, and TT4 and thus MAFLD.

In addition, recent evidence suggests that thyroid dysfunction may not only be associated with hepatic steatosis but also with ectopic fat deposition outside the liver. For example, Bayyigit et al. (Diabetes Metab Res Rev, 2024) reported that both hypothyroidism and subclinical hypothyroidism were significantly associated with an increased prevalence of non-alcoholic fatty pancreas disease (NAFPD) as well as MAFLD. Their findings indicate a potential bidirectional relationship between thyroid dysfunction and ectopic fat accumulation, further underscoring the need for future studies to comprehensively investigate the interplay among hypothyroidism, MAFLD, and NAFPD.

In conclusion, TSH plays a key role in the development and progression of MAFLD and may be influenced by age, which needs to be further elucidated by more clinical trials in different age groups. As thyroid replacement therapy improves elevated TSH levels, whether treating hypothyroidism in patients with MAFLD improves liver function or disease progression outcomes should be further elucidated by placebo-controlled clinical trials.

This study had some limitations. First, heterogeneity was significant in some meta-analyses. We tried to explore heterogeneity between studies through sensitivity and meta-regression analyses and pooled data through random-effects models to obtain more conservative effect estimates. Second, several noninvasive methods [e.g., elevated liver enzymes, fatty liver index, ultrasonography, CT magnetic resonance imaging, and spectroscopy] were commonly used to diagnose MAFLD because liver biopsy was not feasible in the general population (70). While liver biopsy remains the diagnostic gold standard, non-invasive approaches such as the Hepatic Steatosis Index or FibroScan, although practical, may introduce a degree of misclassification bias compared with histology, and this potential bias should be considered when interpreting our results.Third, we did not perform a subgroup analysis of TSH concentration because the upper normal limit of TSH concentration had long been controversial, and the diagnosis of subclinical hypothyroidism depended crucially on the definition of the upper normal limit of TSH concentration (71). We only compared TSH values between the MAFLD and the controls, irrespective of whether the population had hypothyroidism.

## Data Availability

The original contributions presented in the study are included in the article/supplementary material. Further inquiries can be directed to the corresponding authors.
